# Risk factors for rebleeding at different time points after TIPS in cirrhotic patients with AEVB: A case-control study

**DOI:** 10.1097/MD.0000000000048980

**Published:** 2026-05-29

**Authors:** Chunxiu Tan, Wentun Yao, Houqiang Su, Juan Xie, Ruiting Ma, Liya Huang

**Affiliations:** a The First Clinical Medical College, Ningxia Medical University, Yinchuan, China; b Department of Gastroenterology, General Hospital of NingXia Medical University, Yinchuan, China; c Department of Geriatrics, General Hospital of NingXia Medical University, Yinchuan, China.

**Keywords:** liver cirrhosis, risk factors, TIPS, time-point analysis, variceal rebleeding

## Abstract

This retrospective case-control study aimed to identify risk factors for early (≤6 weeks) and late (>2 years) rebleeding after transjugular intrahepatic portosystemic shunt (TIPS) in cirrhotic patients with acute esophagogastric variceal bleeding (AEVB). A total of 208 consecutive patients who underwent TIPS for AEVB at a single tertiary center between January 2021 and December 2023 were enrolled and followed up until May 2025. Multivariate logistic regression was performed to determine independent predictors of rebleeding at different time points. During follow-up (mean 20.06 ± 9.53 months), 12 patients (5.8%) developed early rebleeding, and 21 (10.1%) had late rebeeding. Multivariate analysis showed that elevated preoperative fasting blood glucose (FBG) was an independent protective factor against early rebleeding (OR = 0.861, 95% CI: 0.765–0.969, *P* = .013). For late rebleeding, male gender (OR = 3.820, 95% CI: 1.069–13.651, *P* = .039) and preoperative infection (OR = 6.490, 95% CI: 2.389–17.631, *P = *.001) were identified as independent risk factors. Higher preoperative FBG is associated with lower risk of early rebleeding after TIPS, whereas male gender and preoperative infection predict an increased risk of late rebleeding. These findings may help risk stratification and perioperative management in patients undergoing TIPS for AEVB.

## 1. Introduction

The development of cirrhosis involves a process of hepatocyte necrosis and regeneration, leading to sinusoidal fibrosis and capillarization of the liver. Portal hypertension arises from reduced liver parenchyma, impaired blood flow due to fibrosis and abnormal remodeling, and the formation of portosystemic shunts.^[1]^ Its fundamental pathophysiological basis lies in liver cirrhosis caused by various factors, which leads to a significant increase in the pressure of the portal venous system. This, in turn, forces blood to shunt to the esophagogastric vascular plexus, resulting in the formation of varices. When the hepatic venous pressure gradient (HVPG) exceeds 12 mm Hg, the risk of bleeding increases significantly; if HVPG exceeds 20 mm Hg, it often indicates failure of bleeding control and an elevated mortality rate.^[2]^ Gastrointestinal bleeding, as the most common and severe complication of portal hypertension, is a leading cause of death in cirrhotic patients.^[3]^ Nearly 50% of patients with portal hypertension may develop esophagogastric varices, and among those with existing varices, about one-third will experience acute esophagogastric variceal bleeding (AEVB).^[4]^ Even with standardized treatment, AEVB carries a 30-day mortality rate of 15% to 20%.^[5]^ Transjugular intrahepatic portosystemic shunt (TIPS), established via a minimally invasive approach to create a shunt channel between the hepatic and portal veins within the liver parenchyma, is a key intervention for reducing portal hypertension in cirrhotic patients.^[6]^ However, post-TIPS rebleeding remains a common and serious complication, occurring in 6% to 40% of cases,^[7]^ leading not only to treatment failure but also to higher mortality. While existing studies have explored general risk factors for rebleeding, such as stent dysfunction and portal vein thrombosis^[8]^ most have analyzed rebleeding as a single endpoint without distinguishing between early (within 6 weeks post-TIPS) and late (within 2 years post-TIPS) rebleeding, which may involve distinct pathological mechanisms and risk profiles. Moreover, the influence of potentially modifiable preoperative conditions, such as glycemic metabolic status and infection on rebleeding risk within different time frames has not been adequately elucidated. Based on this clinical observation, this study collected patients who underwent TIPS for AEVB at the General Hospital of Ningxia Medical University between January 2021 and December 2023. Through a case-control design, it aimed to specifically investigate the independent risk factors for early (≤6 weeks) and late (>2 years) rebleeding after TIPS in cirrhotic patients with AEVB, with the goal of providing clinical references for identifying high-risk patients and implementing individualized preoperative interventions and postoperative monitoring.

## 2. Materials and methods

### 2.1. Ethics

This study has been approved by the Ethics Committee of the General Hospital of Ningxia Medical University (Approval No. KYLL-2021-859; Approval Date: January 1, 2022). All patients signed the informed consent form.

### 2.2. Patients

A total of 208 patients who underwent TIPS for AEVB at the Department of Gastroenterology, General Hospital of Ningxia Medical University between January 2021 and December 2023 were included. Patients were followed up for at least 12 months (mean [SD] duration: 20.06 ± 9.53 months) until May 2025. The cohort consisted of 135 males (64.9%) and 73 females (35.1%), with an age range of 27 to 74 years (mean [SD]: 54.75 ± 10.65 years).

#### 2.2.1. Inclusion criteria

1. Aged > 18 years;
2. Meets the diagnostic criteria for liver cirrhosis with portal hypertension complicated by AEVB;
3. All patients received TIPS treatment.

#### 2.2.2. Exclusion criteria

1. Patients with gastrointestinal bleeding caused by other sites or reasons;
2. Patients with severe cardiopulmonary diseases, multiple organ failure, and those unable to cooperate with endoscopy or TIPS.

### 2.3. Definition of preoperative infection

Preoperative infection was diagnosed based on clinical manifestations, imaging findings, and elevated inflammatory markers (CRP, PCT), including spontaneous bacterial peritonitis, respiratory tract infection, urinary tract infection, and biliary infection.

### 2.4. Data collection and follow-up

Patient data were retrospectively collected. Baseline characteristics included age, gender, etiology of liver cirrhosis, and the presence of specific comorbidities (hypertension, diabetes, liver cancer), along with portal vein thrombosis, ascites, and preoperative infection. Laboratory parameters assessed immediately prior to TIPS comprised: red blood cell count (RBC), white blood cell count (WBC), hemoglobin (Hb), and platelet count (PLT) for hematologic profile; prothrombin activity (PTA) and international normalized ratio (INR) for coagulation status; aspartate aminotransferase (AST), alanine aminotransferase (ALT), alkaline phosphatase (ALP), γ-glutamyl transpeptidase (GGT), total bilirubin (TBIL), and albumin (ALB) for liver function; serum creatinine (Cr) for renal function; and FBG.

### 2.5. Statistical analysis

Statistical analysis was performed using SPSS 25.0 software (IBM Corporation). Continuous variables were described using the mean ± standard deviation (SD) and comparisons between 2 groups were performed using the independent samples *t* test. Continuous data with a non-normal distribution were presented as M(P25–P75), and inter-group comparisons were conducted using the rank-sum test. Categorical data were expressed as case numbers (%), and inter-group comparisons were carried out using the chi-square test (χ^2^ test) or Fisher exact test. Multivariate logistic regression analysis was used to identify the risk factors for rebleeding after TIPS. *P* < .05 was considered statistically significant.

## 3. Results

### 3.1. Baseline characteristics

A total of 208 patients were enrolled in this study. The mean age was 54.75 ± 10.65 years (range: 27–74), with 135 males (64.9%) and 73 females (35.1%). The primary etiology of cirrhosis was hepatitis B virus infection (143 cases, 68.8%), followed by autoimmune liver disease (26 cases, 12.5%), cryptogenic cirrhosis (23 cases, 11.1%), hepatitis C virus infection (6 cases, 2.9%), and alcoholic liver disease (3 cases, 1.4%); other causes (e.g., polycystic liver disease, drug-induced cirrhosis) accounted for 7 cases (3.4%). Preoperative comorbidities included diabetes mellitus (37 cases, 17.8%), hepatocellular carcinoma (30 cases, 14.4%), and hypertension (28 cases, 13.5%). All patients with hepatitis B or C virus infection received standard antiviral therapy. Regarding the treatment pathway, 100 patients (48.1%) underwent TIPS directly following AEVB, while the remaining 108 patients (51.9%) received salvage TIPS after failed initial endoscopic sclerotherapy or variceal ligation. All TIPS procedures were performed by a single operator to minimize bias.

### 3.2. Analysis of risk factors for rebleeding within 6 weeks after TIPS

Patients were categorized into 2 groups depending on whether rebleeding occurred within 6 weeks after TIPS, the rebleeding group, comprising 12 cases (5.77%), and the non-rebleeding group, comprising 196 cases (94.23%). A comparison was conducted between the 2 groups regarding general patient characteristics, laboratory parameters, Child–Pugh liver function scores, comorbidities, and follow-up data to evaluate statistically significant differences (Table 1). As presented in Table 1, the preoperative FBG level was the only variable that exhibited a statistically significant difference (*P* < .05) between the groups. The FBG level in the rebleeding group (median 10.58 mmol/L; IQR 6.69–12.64) was significantly higher than that in the non-rebleeding group (median 6.42 mmol/L; IQR 5.04–8.02). No statistically significant differences were observed in the other factors analyzed.

**Table 1:** Univariate analysis of factors associated with rebleeding within 6 weeks after TIPS.

Characteristics	Rebleeding group (n = 12)	Non-rebleeding group (n = 196)	χ^*2*^*/t*	*P*
Average age, yr	58.42 ± 10.27	54.53 ± 10.65	0.45	.22
Gender, n (%)			0.57	.45
Male	9 (75%)	126 (64.29%)		
Female	3 (25%)	70 (35.71%)		
Cause, n (%)			5.217	.266
Post-hepatitis B/C cirrhosis	7 (58.33%)	142 (72.46%)		
Autoimmune hepatitis (AIH)	4 (33.34%)	22 (11.22%)		
Unknown etiology	1 (8.33%)	22 (11.22%)		
Alcoholic liver disease (ALD)	0	3 (1.53%)		
Other	0	7 (3.57%)		
With preoperative infection, n (%)	6 (50%)	135 (68.88%)	1.082	.298
With ascites	8 (66.67%)	126 (64.29%)	–	.867*
With liver cancer, n (%)	2 (16.67%)	28 (14.29%)	–	1.000*
With thrombosis, n (%)	4 (33.33%)	59 (30.10%)	–	1.000*
Blood index				
WBC [M(P25–P75)], ×10^9^/L	5.18 (2.16, 6.56)	3.21 (1.96, 5.37)	−1.71	.087
RBC (*X* ± *S*), ×10^9^/L	2.30 ± 0.83	3.08 ± 0.61	2.51	.734
Hb [M(P25–P75)], g/L	79.00 (57.00, 107.00)	83.00 (75.00, 96.00)	−0.03	.976
PLT [M(P25–P75)], ×10^9^/L	58.00 (28.00, 86.00)	56.50 (42.00, 80.00)	−0.497	.619
ALB (*X* ± *S*), g/L	30.46 ± 5.06	31.94 ± 4.89	0.104	.31
PTA (*X* ± *S*), %	59.92 ± 16.42	63.84 ± 13.46	0.528	.433
INR (*X* ± *S*)	1.50 ± 0.32	1.40 ± 0.22	4.136	.317
NA (*X* ± *S*), mmol/L	139.05 ± 2.46	139.97 ± 3.85	2.825	.415
AST [M(P25–P75)], U/L	29.60 (23.70, 40.30)	26.90 (20.03, 36.30)	−1.364	.173
ALT [M(P25–P75)], U/L	23.70 (16.20, 33.10)	23.80 (16.38, 33.95)	−0.351	.726
ALP [M(P25–P75)], U/L	85.80 (42.00, 123.00)	66.15 (50.00, 86.83)	−1.188	.235
GGT [M(P25–P75)], U/L	34.60 (14.00, 109.20)	27.90 (18.05, 48.98)	−0.284	.776
Ammonia [M(P25–P75)], μmol/L	45.00 (43.70, 62.00)	43.00 (27.00, 51.75)	−1.578	.115
FBG [M(P25–P75)], mmol/L	10.58 (6.69, 12.64)	6.42 (5.04, 8.02)	−2.675	.007
Cr [M(P25–P75)], μmol/L	63.90 (48.60, 83.20)	60.30 (50.71, 71.98)	−0.941	.347
Ur [M(P25–P75)], mmol/L	8.57 (3.82, 9.52)	5.66 (3.87, 8.40)	−0.926	.354
TBIL [M(P25–P75)], μmol/L	34.60 (13.00, 56.70)	22.79 (15.90, 31.65)	−1.257	.209
LOS [M(P25–P75)], d	13.00 (9.00, 20.00)	11.00 (8.00, 14.00)	−1.627	.100
MELD score [M(P25–P75)]	13.00 (10.00, 16.00)	12.00 (10.00, 14.00)	−1.037	.300
Child–Pugh class (A/B/C)	02-07-03	57/104/35	1.074	.610
Diabetes, n (%)	3 (25%)	34 (17.35%)	0.081	.776
Hypertension, n (%)	1 (8.33%)	27 (13.78%)	0.010	.920

AIH = autoimmune hepatitis, ALB = albumin, ALD = alcoholic liver disease, ALP = alkaline phosphatase, ALT = alanine aminotransferase, AST = aspartate aminotransferase, Cr = creatinine, FBG = fasting blood glucose, GGT = γ-glutamyl transpeptidase, Hb = hemoglobin, INR = international normalized ratio, LOS = length of hospital stay, MELD = model for end-stage liver disease, NA = serum sodium, PLT = platelet, PTA = prothrombin activity, RBC = red blood cell, TBIL = total bilirubin, TIPS = transjugular intrahepatic portosystemic shunt, WBC = white blood cell.

* Fisher exact test was used for statistical calculation.

Multivariate logistic regression was performed incorporating factors significantly associated (*P* < .05) with rebleeding within 6 weeks in the univariate analysis (Table 2). The model revealed that a higher preoperative FBG level was an independent protective factor against rebleeding. For each 1 mmol/L increment in FBG, the risk of rebleeding was reduced by 13.9% (OR = 0.861, 95% CI: 0.765–0.969, *P = *.013).

**Table 2:** Multivariate analysis for rebleeding within 6 weeks post-TIPS.

Factor	*B* value	SE	WALD	OR	95% CI	*P* value
FBG (mmol/L)	−0.150	0.060	6.189	0.861	0.765–0.969	.013
Constant	4.070	0.659	30.436	0.006	–	–

CI = confidence interval, FBG = fasting blood glucose, OR = odds ratio, SE = standard error, TIPS = transjugular intrahepatic portosystemic shunt.

### 3.3. Analysis of risk factors for rebleeding at >2 years after TIPS

A total of 21 out of 208 patients (10.10%) experienced rebleeding within 2 years after the TIPS procedure, constituting the rebleeding group, while the remaining 187 patients (89.90%) comprised the non-rebleeding group. The univariate analysis comparing these groups demonstrated significant differences in gender, FBG, and preoperative concurrent infection status (*P* < .05 for all; Table 3). Indicators showing significance in the univariate analysis were entered into a multivariate logistic regression model to identify independent risk factors. The results confirmed that male gender (OR = 3.820, 95% CI: 1.069–13.651, *P* = .039) and the presence of a preoperative concurrent infection remained independently associated with an increased risk of rebleeding within 2 years after TIPS (Table 4).

**Table 3:** Comparison of patient characteristics between the rebleeding and non-rebleeding groups within 2 years after TIPS.

Characteristics	Rebleeding group (n = 21)	Non-rebleeding group (n = 187)	χ^*2*^*/t*	*P* value
Average age, yr	52.24 ± 9.92	55.03 ± 10.71	0.296	.255
Gender, n (%)			4.441	.035
Male	18 (85.71%)	117 (62.57%)		
Female	3 (14.29%)	70 (37.43%)		
Cause, n (%)			6.641	.156
Post-hepatitis B/C cirrhosis	20 (95.24%)	129 (68.98%)		
AIH	1 (4.76%)	25 (13.37%)		
Unknown etiology	0	23 (12.30%)		
ALD	0	3 (1.60%)		
Other	0	7 (3.75%)		
Preoperative infection, n (%)	6 (28.57%)	135 (72.19%)	16.452	.001
With ascites, n (%)	16 (76.19%)	114 (60.96%)	1.868	.172
With liver cancer, n (%)	2 (9.52%)	28 (14.97%)	0.120	.729
With thrombosis, n (%)	6 (28.57%)	57 (30.48%)	0.033	.857
Blood index				
WBC [M(P25–P75)], ×10^9^/L	3.63 (2.48, 5.87)	3.23 (1.97, 5.43)	−0.522	.602
RBC (*X* ± *S*), ×10^9^/L	3.02 ± 0.87	3.08 ± 0.59	2.51	.734
HB (*X* ± *S*), g/L	85.52 ± 28.82	86.47 ± 18.40	5.702	.884
PLT [M(P25–P75)], ×10^9^/L	52.00 (41.50, 80.50)	57.00 (41.00, 80.00)	−0.076	.939
ALB (*X* ± *S*), g/L	32.47 ± 4.25	31.79 ± 4.97	0.086	.549
PTA (*X ± S*), %	61.67 ± 12.87	63.83 ± 13.73	0.308	.492
INR (*X ± S*)	1.44 ± 0.22	1.40 ± 0.23	0.175	.485
NA (*X* ± *S*), mmol/L	139.47 ± 3.70	139.97 ± 3.80	0.061	.566
AST [M(P25–P75)], U/L	21.50 (18.25, 31.05)	28.50 (20.90, 37.00)	−1.964	.050
ALT [M(P25–P75)], U/L	24.40 (15.10, 35.90)	23.80 (16.20, 33.80)	−0.205	.838
ALP [M(P25–P75)], U/L	61.80 (53.35, 84.50)	69.00 (50.00, 89.00)	−0.180	.857
GGT [M(P25–P75)], U/L	28.70 (17.00, 57.15)	28.10 (17.90, 49.00)	−0.065	.948
Ammonia [M(P25–P75)], μmol/L	43.69 (30.50, 69.50)	43.69 (27.00, 51.00)	−1.211	.226
FBG [M(25–P75)]/mmol/L	8.22 (5.40, 12.17)	6.45 (5.09, 7.76)	−2.055	.040
Cr [M(P25–P75)], μmol/L	57.50 (52.60, 63.65)	60.40 (50.30, 73.00)	−0.855	.393
Ur [M(P25–P75)], mmol/L	5.96 (5.07, 8.23)	5.67 (3.76, 8.57)	−0.933	.351
TBIL [M(P25–P75)], μmol/L	21.31 (16.75, 29.70)	22.90 (15.67, 34.41)	−0.401	.688
LOS [M(P25–P75)], d	9.00 (8.00, 14.00)	11.00 (8.00, 14.00)	−1.004	.316
MELD Class [M(P25–P75)]	12.00 (11.00, 14.00)	12.00 (10.00, 14.00)	−0.900	.928
Child–Pugh Class (A/B/C)	05-12-04	54/99/34	1.074	.610
With diabetes, n (%)	6 (28.57%)	31 (16.58%)	1.128	.288
With hypertension, n (%)	3 (14.29%)	25 (13.37%)	–	.100*

AIH = autoimmune hepatitis, ALB = albumin, ALD = alcoholic liver disease, ALP = alkaline phosphatase, ALT = alanine aminotransferase, AST = aspartate aminotransferase, Cr = creatinine, FBG = fasting blood glucose, GGT = γ-glutamyl transpeptidase, Hb = hemoglobin, INR = international normalized ratio, LOS = length of hospital stay, MELD = model for end-stage liver disease, NA = serum sodium, PLT = platelet, PTA = prothrombin activity, RBC = red blood cell, TBIL = total bilirubin, TIPS = transjugular intrahepatic portosystemic shunt, WBC = white blood cell.

* Fisher exact test was used for statistical calculation.

**Table 4:** Multivariate analysis for rebleeding within 2 years post-TIPS.

Factor	*B* value	SE	WALD	OR	95% CI	*P* value
Gender	1.340	0.650	4.255	3.820	1.069–13.651	.039
FBG	0.100	0.059	2.893	1.105	0.801–1.987	.089
Preoperative infection	1.870	2.328	13.456	6.490	2.389–17.631	.001
Constant	−1.677	0.603	7.741	0.187	–	–

CI = confidence interval, FBG = fasting blood glucose, OR = odds ratio, SE = standard error, TIPS = transjugular intrahepatic portosystemic shunt.

## 4. Discussion

AEVB remains one of the most life-threatening complications of portal hypertension in cirrhosis, carrying high short-term mortality despite advances in endoscopic and interventional management.^[9]^ TIPS effectively reduces portal pressure and controls acute bleeding, yet post-procedural rebleeding still occurs and impairs long-term outcomes. Most previous studies analyzed rebleeding as a single endpoint rather than distinguishing early and late events, which may mask distinct pathophysiological mechanisms and predictors. The present study focused on risk factors for early (≤6 weeks) and late (>2 years) rebleeding separately, providing time-stratified evidence for clinical practice. AEVB remains one of the most life-threatening complications of portal hypertension in cirrhosis, carrying high short-term mortality despite advances in endoscopic and interventional management. Among patients with cirrhosis, esophageal varices occur in 50% to 60% of cases, a prevalence that rises sharply to 85% in those with decompensated cirrhosis. Furthermore, the 6-week mortality risk for these patients is as high as 15% to 30%, and this risk is particularly elevated when infection is present.^[10,11]^ TIPS effectively reduces portal pressure and controls acute bleeding, yet post-procedural rebleeding still occurs and impairs long-term outcomes. Most previous studies analyzed rebleeding as a single endpoint rather than distinguishing early and late events, which may mask distinct pathophysiological mechanisms and predictors. The present study focused on risk factors for early (≤6 weeks) and late (>2 years) rebleeding separately, providing time-stratified evidence for clinical practice.

In our cohort, the 6-week rebleeding rate was 5.77% and the 2-year rebleeding rate was 10.10%, consistent with previously reported ranges of 3% to 25% in the literature.^[12-14]^ Early rebleeding is commonly attributed to acute stent thrombosis, incomplete variceal embolization, or residual portosystemic collaterals (consistent with reported rates of 5–10%^[15]^), while late rebleeding is mostly related to shunt stenosis, occlusion, or recurrent portal hypertension (aligning with rates of approximately 10–15%^[16]^). Our time-dependent design allowed us to capture predictors specific to these 2 phases.

A novel finding of this study is that an elevated preoperative FBG level serves as an independent protective factor against early rebleeding after TIPS (OR = 0.861, 95% CI: 0.765–0.969). Although this result appears paradoxical to the conventional understanding that diabetes generally increases the risk of bleeding in cirrhotic patients, the underlying mechanism can be reasonably explained: acute perioperative stress hyperglycemia may transiently enhance platelet activity and coagulation function,^[17,18]^ thereby helping to maintain the fragile hemostatic balance in the early post‑TIPS period. This finding does not imply that hyperglycemia is clinically beneficial, but rather suggests that overly stringent glycemic control should be avoided in the early postoperative phase to prevent hypoglycemia. For reducing the risk of early rebleeding after TIPS, maintaining stable blood glucose levels may be more critical than intensive glucose‑lowering therapy.

Our study further confirmed that male sex is an independent risk factor for late rebleeding after TIPS, a finding consistent with previous large-scale observational studies.^[19]^ The underlying biological mechanisms that may explain this gender disparity are multifactorial. Primarily, hormonal influences play a crucial role. Androgen-mediated upregulation of vascular endothelial growth factor (VEGF) promotes pathological neoangiogenesis, leading to the formation of fragile and unstable collateral vessels that are prone to rupture.^[20]^ Concurrently, males have a relative deficiency in the endothelial protective effects conferred by estrogen, which in females enhances vascular repair and stability through nitric oxide (NO)-mediated vasodilation and anti-inflammatory signaling.^[20,21]^ This dual hormonal imbalance-excess pro-angiogenic and pro-inflammatory drive coupled with deficient protective signaling creates a vascular microenvironment that is less resilient to hemodynamic stress and more susceptible to chronic injury.

Beyond intrinsic biological factors, modifiable behavioral patterns prevalent in male populations significantly contribute to the elevated risk. Male patients demonstrate higher rates of smoking and alcohol consumption, both of which are well-established risk factors for systemic endothelial dysfunction and the exacerbation of chronic inflammation. Smoking induces direct endothelial injury, promotes oxidative stress, and accelerates atherosclerosis, which can affect the splanchnic and systemic vasculature. Chronic alcohol use, independent of alcoholic liver disease etiology, can further impair hepatic sinusoidal endothelial cell function and portal hemodynamics. These behaviors collectively aggravate endothelial injury and sustain a pro-inflammatory state, which can impede proper stent endothelialization, promote shunt stenosis or thrombosis, and ultimately impair long-term shunt patency, thereby increasing the susceptibility to late rebleeding events.

Preoperative infection was identified as another strong independent predictor of late rebleeding following TIPS, underscoring its critical role in long-term therapeutic failure. The underlying pathophysiological link can be conceptualized as a self-perpetuating Infection–Inflammation–Portal Hypertension vicious cycle. The cycle is often initiated by intestinal bacterial translocation, a common event in cirrhosis due to gut barrier dysfunction. The released pathogen-associated molecular patterns, particularly lipopolysaccharide (LPS), activate the Toll-like receptor 4 (TLR-4) signaling pathway in hepatic immune cells (e.g., Kupffer cells) and stellate cells. This activation triggers a robust cascade of pro-inflammatory cytokines, including tumor necrosis factor-α (TNF-α) and interleukin-6 (IL-6).^[22]^

These inflammatory mediators exert multiple detrimental effects: they directly inflict damage on liver sinusoidal endothelial cells, disrupt normal hepatic microcirculation, and stimulate hepatic stellate cells to promote intrahepatic fibrosis and pathological angiogenesis. Collectively, these processes increase intrahepatic vascular resistance, thereby elevating portal pressure – a fundamental driver of variceal rebleeding.^[23]^ Beyond modulating portal hemodynamics, infection critically disrupts the fragile coagulation balance in cirrhosis. Patients with cirrhosis exist in a state of rebalanced hemostasis, which is easily tipped toward either bleeding or thrombosis. Systemic inflammation from infection can cause endothelial dysfunction, activate platelets, and simultaneously consume clotting factors, creating a pro-thrombotic yet bleeding-prone state. This dysregulated milieu significantly increases the risk of stent thrombosis or endothelialization failure, directly compromising shunt patency.

Furthermore, severe infections, such as septic shock, induce profound hemodynamic disturbances – including systemic vasodilation and reduced effective arterial blood volume – which can paradoxically further aggravate portal hypertension by increasing splanchnic blood flow.^[24]^ Thus, infection not only provides the initial insult but also creates a feed-forward loop where elevated portal pressure and inflammation sustain each other, culminating in recurrent variceal hemorrhage.

These compelling mechanistic insights translate into a clear and urgent clinical imperative: rigorous preoperative screening for and active management of infections are not merely supportive measures but are integral to the success of TIPS. A comprehensive preoperative workup should include meticulous clinical evaluation, targeted imaging, and assessment of inflammatory markers (e.g., CRP, procalcitonin) to identify and treat conditions like spontaneous bacterial peritonitis, pneumonia, or urinary tract infections before TIPS placement. Effectively breaking this “Infection–Inflammation–Portal Hypertension” cycle prior to intervention is likely a key strategy for improving long-term shunt patency and reducing the burden of late rebleeding.

This study has several limitations. First, it is a single-center retrospective design with a relatively small sample size, particularly in the early rebleeding group (n = 12), which may limit statistical power. Second, preoperative infection was defined based on clinical and medication records rather than standardized microbiological criteria, which may introduce classification bias. Third, technical parameters of TIPS (stent type, diameter, pressure gradient reduction) were not analyzed, although they are known to affect rebleeding. Finally, selection and information biases are inherent in retrospective research. Future large‑sample, multicenter, prospective studies are warranted to validate these findings and adjust for procedural variables.

In summary, preoperative FBG, sex, and infection status show time‑dependent effects on rebleeding after TIPS for AEVB. Higher FBG protects against early rebleeding, while male sex and preoperative infection increase the risk of late rebleeding. These biomarkers can be used for risk stratification, perioperative optimization, and personalized surveillance strategies in clinical practice.

## Author contributions

**Conceptualization:** Chunxiu Tan, Liya Huang.

**Data curation:** Wentun Yao, Houqiang Su, Ruiting Ma.

**Formal analysis:** Houqiang Su.

**Funding acquisition:** Wentun Yao, Ruiting Ma.

**Investigation:** Juan Xie.

**Software:** Juan Xie.

**Visualization:** Liya Huang.
